# Mth10b, a Unique Member of the Sac10b Family, Does Not Bind Nucleic Acid

**DOI:** 10.1371/journal.pone.0019977

**Published:** 2011-05-18

**Authors:** Yan-Feng Liu, Nan Zhang, Hong-Wei Yao, Xian-Ming Pan, Meng Ge

**Affiliations:** 1 Ministry of Education Key Laboratory of Bioinformatics, School of Life Sciences, Tsinghua University, Beijing, People's Republic of China; 2 National Laboratory of Biomacromolecules, Center for Structure and Molecular Biology, Institute of Biophysics, Chinese Academy of Sciences, Beijing, People's Republic of China; University of Oldenburg, Germany

## Abstract

The Sac10b protein family is regarded as a group of nucleic acid-binding proteins that are highly conserved and widely distributed within archaea. All reported members of this family are basic proteins that exist as homodimers in solution and bind to DNA and/or RNA without apparent sequence specificity *in vitro*. Here, we reported a unique member of the family, Mth10b from *Methanobacterium thermoautotrophicum ΔH*, whose amino acid sequence shares high homology with other Sac10b family proteins. However, unlike those proteins, Mth10b is an acidic protein; its potential isoelectric point is only 4.56, which is inconsistent with the characteristics of a nucleic acid-binding protein. In this study, Mth10b was expressed in *Escherichia coli* and purified using a three-column chromatography purification procedure. Biochemical characterization indicated that Mth10b should be similar to typical Sac10b family proteins with respect to its secondary and tertiary structure and in its preferred oligomeric forms. However, an electrophoretic mobility shift analysis (EMSA) showed that neither DNA nor RNA bound to Mth10b *in vitro*, indicating that either Mth10b likely has a physiological function that is distinct from those of other Sac10b family members or nucleic acid-binding ability may not be a fundamental factor to the actual function of the Sac10b family.

## Introduction

Sac10b, generally regarded as a chromosomal DNA-binding protein, was first isolated from the hyperthermophile *Sulfolobus acidocaldarius* by Grote *et al.* in the 1986 [1]. After an initial routine biochemical characterization [1]–[3], study of the protein lagged until 1999, when Forterre *et al.* found that the gene *sac10b*, which encodes Sac10b, has one or two homologs in each of the archaeal organisms whose genomes had been sequenced [4]. The proteins encoded by these genes were classified as members of the Sac10b protein family. The ubiquity of these proteins suggests that they play an important physiological role in Archaea [4]. In recent years, typical proteins from this family, including Ssh10b from *Sulfolobus shibatae* [5]–[14] and Sso10b and Sso10b2 from *Sulfolobus solfataricus* [15]–[21], have been studied extensively.

Sac10b binds cooperatively to DNA with no significant compaction and protects DNA against degradation by the nuclease DNase I *in vitro* [2]. However, electron microscopy of immunogold-stained Sac10b has shown that it is present primarily in the cytoplasm *in vivo* [3]. Ssh10b binds to double-stranded DNA, single-stranded DNA and RNA with similar affinities *in vitro* [5], [6] and affects DNA topology in a temperature-dependent manner [5], [7]. *In vivo* UV cross-linking and co-immunoprecipitation experiments using *S. shibatae* cells, however, have shown that Ssh10b binds exclusively to RNA [6]. Sso10b also binds to DNA and RNA with no apparent sequence specificity *in vitro* [15]–[17], but it is reversibly acetylated at a single lysine residue *in vivo*, which results in a reduction in its nucleic acid-binding affinity [15]–[17]. As is true for the histones of eukaryotic chromatin, acetylation and deacetylation of Sso10b is thought to play an important role in chromatin regulation. However, chromatin immunoprecipitation results imply that Sso10b is associated with both types of nucleic acids *in vivo* [17].

In 2002, the crystal structure of Sso10b was determined by Wardleworth *et al.* [16]. Sso10b is a small, basic protein that forms a homodimer in solution. The crystal structure of Sso10b revealed that the monomer has a mixed α/β fold comprising two α-helices and four β-sheets and resembles that of the C-terminal domain of the bacterial translation initiation factor IF3 [16]. The shape of the Sso10b dimer resembles a body with two long, outstretched β-hairpin arms that can be docked onto a DNA duplex, where they contact equivalent minor groove regions and allow the highly basic central body to contact the major groove [16]. Then in the following several years, the structures of various other Sac10b proteins, including Sso10b2 from *Sulfolobus solfataricus* [18], [19], Ssh10b from *Sulfolobus shibatae* [7], Mja10b from *Methanocaldococcus jannaschii* [22], Afu10b from *Archaeoglobus fulgidus* [23], Ape10b2 from *Aeropyrum pernix K1* [24], and Pho10b from *Pyrococcus horikoshii OT3* [25], have been solved in succession. They are all small, basic homodimeric proteins with a highly superimposable β_1_α_1_β_2_α_2_β_3_β_4_ topology. Although the lengths of the flexible β-hairpin arms vary, the central presumed DNA-binding surfaces are all distributed with positively charged residues predominantly.

**Figure 1:**
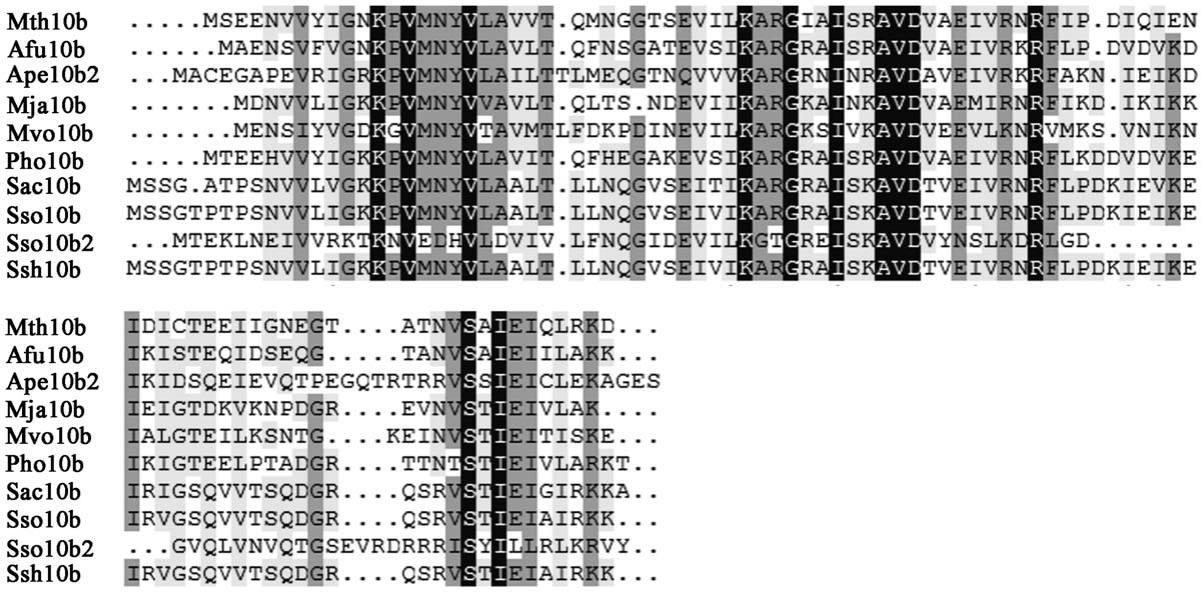
Sequence alignment of Mth10b and those reported typical members of Sac10b protein family: Mth (*Methanobacterium thermoautotrophicum*), Afu (*Archaeoglobus fulgidus*), Ape (*Aeropyrum pernix K1*), Mja (*Methanocaldococcus jannaschii*), Mvo (Methanococcus voltae), Pho (*Pyrococcus horikoshii*), Sac (*Sulfolobus acidocaldarius*), Sso (*Sulfolobus solfataricus*), Ssh (*Sulfolobus shibatae*). The figure was rendered using the program DNAMAN.

The most extensively studied methanogen, *Methanobacterium thermoautotrophicum ΔH*, is a lithoautotrophic, thermophilic archaeon that grows at temperatures in the range of 40–70°C, with an optimal temperature of 65°C [26]. Like most Archaea, its genome also includes a member of the Sac10b protein family, Mth10b, whose amino acid sequence shares high homology with typical Sac10b protein family members (Figure 1). However, unlike other Sac10b proteins, Mth10b is an acidic protein with an isoelectric point of 4.56 (Table 1), which is inconsistent with the characteristics of a nucleic acid-binding protein. An interesting question therefore arises: is Mth10b a nucleic acid-binding protein?

**Table 1:** Comparison of numbers of acidic and basic residues in Mth10b and other Sac10b family members.

Name	Acidic residues	Basic residues	Potential Isoelectric point
Mth10b	***13***	***8***	***4.56***
Afu10b	11	12	8.27
Ape10b2	14	17	9.10
Mja10b	11	16	9.65
Mvo10b	12	13	8.15
Pho10b	14	15	8.23
Sac10b	8	16	10.48
Sso10b	8	16	10.48
Sso10b2	13	16	9.40
Ssh10b	8	16	10.48

Their potential isoelectric points are also listed in the table.

To answer this question, we cloned and expressed Mth10b in *Escherichia coli* cells and developed an efficient protocol for Mth10b purification. Biochemical characterization suggested that Mth10b has a structure similar to the structures of other typical Sac10b proteins. However, an electrophoretic mobility shift analysis (EMSA) showed that neither DNA nor RNA bound to Mth10b *in vitro*, suggesting that either Mth10b likely has a physiological function that is distinct from those of other Sac10b family members or nucleic acid-binding ability may not be a fundamental factor to the actual function of the Sac10b family.

## Results

### Identification of the *mth10b* gene

The amino acid sequence of Sso10b, a typical member of the Sac10b protein family, was used to perform a BLAST search [27] in genebank. After analyzing the amino acid compositions of the Sac10b family proteins returned by the search, we found a unique putative protein from *Methanobacterium thermoautotrophicum ΔH*, encoded by the gene MTH1483, which we termed Mth10b. The amino acid sequence of Mth10b shares identities with Sso10b, Sso10b2, Ssh10b, Mja10b, Afu10b, Ape10b2, and Pho10b of 50.5, 30.9, 50.5, 56.0, 61.5, 45.1, and 61.3%, respectively (Figure 1), indicating the protein may have a structure similar to those of typical Sac10b family proteins. However, unlike all reported members of the Sac10b family, Mth10b is an acidic protein. Table 1 lists the acidic residue numbers, the basic residue numbers and potential isoelectric points of Mth10b and other seven reported members of the Sac10b protein family. As shown in the table, all those typical Sac10b family members are basic proteins with potential isoelectric point higher than 8 and have more basic amino acid residues than acidic ones. While Mth10b has many more acidic amino acid residues (13) than basic ones (8), and its potential isoelectric point is only 4.56, which is inconsistent well with the characteristics of a nucleic acid-binding protein.

### Protein expression and purifaction

**Figure 2:**
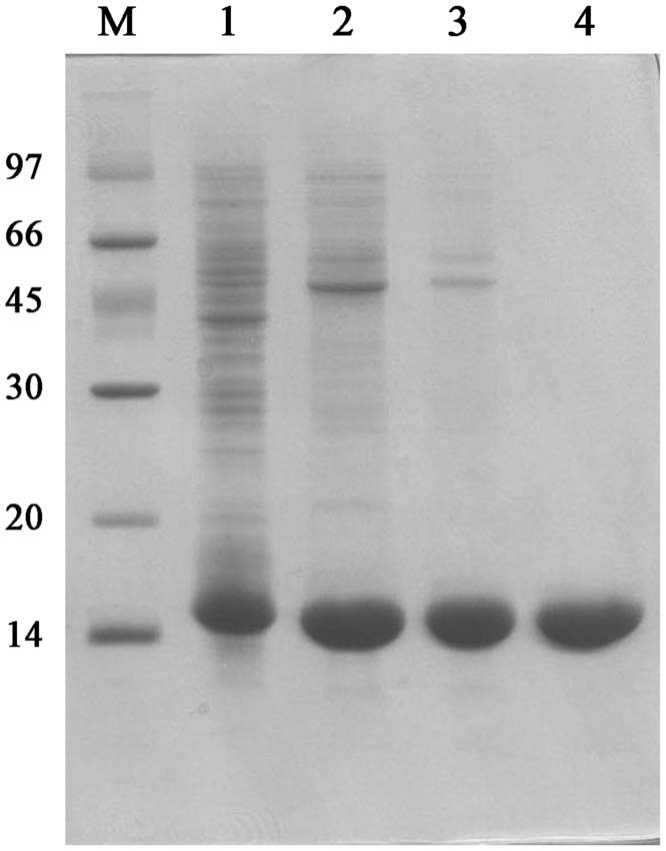
SDS-PAGE analysis of Mth10b expression and purification in *Escherichia coli*: lane 1, low molecular weight marker; lane 2, total cell lysate after induction; lane 3, samples purified after Q Sepharose Fast Flow column; lanes 4, samples purified after Phenyl Sepharose High Performance column; lanes 5, purified Mth10b after Resource-S column.

The *mth10b* gene was cloned into the expression vector pET11a and expressed in *Escherichia coli* BL21 (DE3) cells as described in methods. Analysis by 15% SDS-PAGE (Figure 2) showed that it has an apparent molecular weight of approximately 14 kDa. This size is slightly larger than expected, an effect that could be explained by the abnormal distribution of negatively charged residues in Mth10b. When the SDS-PAGE gel was scanned and analyzed by Scion Image, Mth10b constituted approximately 30% of the total protein.

For purification of recombinant thermophilic or hyperthermophilic proteins in *Escherichia coli*, maintaining cells lysate at high temperature for several minutes is usually an efficient method to precipitate those unwanted proteins. However, though *Methanobacterium thermoautotrophicum ΔH* grows optimally at 65°C, the recombinant protein Mth10b was precipitated absolutely after the cells lysate was maintained at 60°C for 20 minutes. This phenomenon suggested that some external factors contribute to the thermostability of Mth10b in *Methanobacterium thermoautotrophicum* cells.

**Figure 3:**
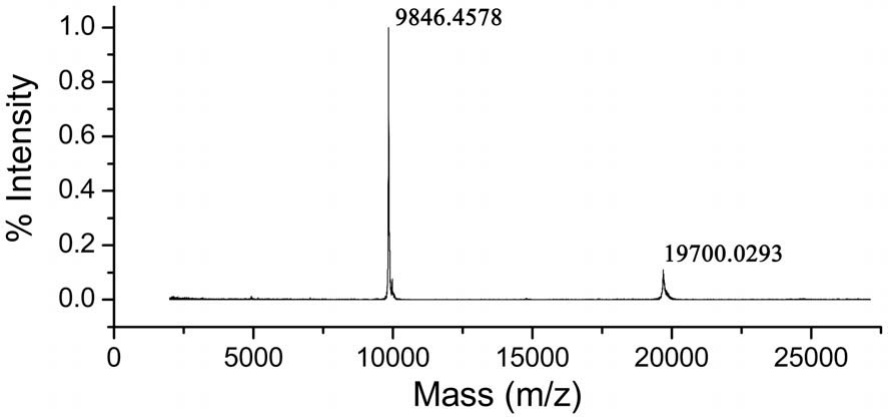
MALDI-TOF mass spectrum of purified Mth10b recorded on an ABI 4800 plus MALDI-TOF-TOF mass spectrometry.

Cells containing the target protein were lysed by ultrasonication, and the supernatant was subjected to the three-column chromatography purification procedure described in Methods to yield a homogeneous product (Figure 2). Protein purity was greater than 95%, as confirmed by 15% SDS-PAGE. MALDI-TOF mass spectrometry revealed a mass of 9846.48 D (Figure 3), in agreement with the calculated molecular mass of Mth10b lacking the N-terminal methionine residue. N-terminal sequencing of the purified protein confirmed its identity (data not shown).

### Secondary structure of Mth10b

**Figure 4:**
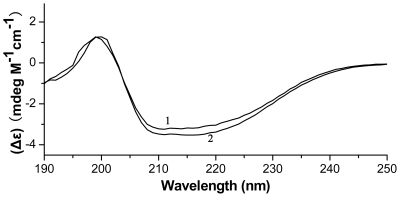
Far-UV CD spectra of Mth10b and Ssh10b in buffer F recorded at 25°C: line 1, the spectrum of Ssh10b; line 2, the spectrum of Mth10b.

Far-UV circular dichroism (CD) is a common method to study protein secondary structure because different types of regular secondary structure found in proteins give rise to characteristic CD spectra in the far UV region. Using Ssh10b, a typical member of the Sac10b family, as a control, we investigated the secondary structure of Mth10b with far-UV CD spectroscopy. The far-UV CD spectrum of Mth10b, recorded at pH 7.5 and 25°C and presented in Figure 4, reveals a typical spectrum of a mixed α/β structure. As shown in the Figure, the overall shape of the spectrum is very similar with that of Ssh10b, which is consistent with that of previous reported [5], [8], [14]. The spectrum of Mth10b is also similar to those of other typical members of the Sac10b family, including Pho10b [25], and Mvo10b [28]. These results suggested that the secondary structure of Mth10b is similar to those of typical members of the Sac10b family.

### Tertiary structure of Mth10b

**Figure 5:**
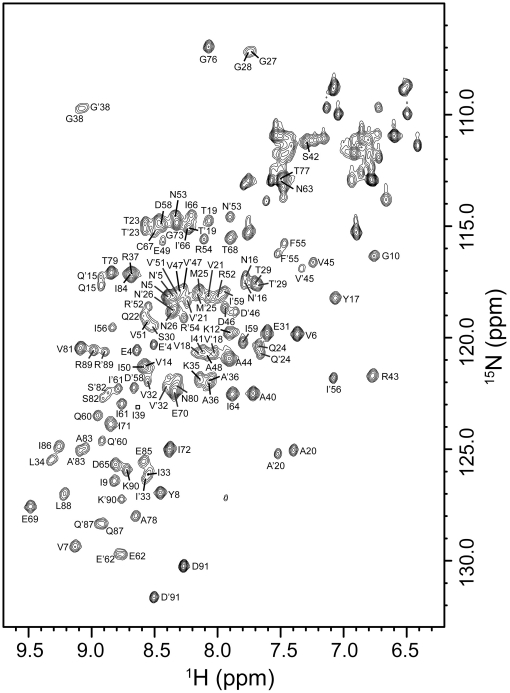
2D ^1^H-^15^N HSQC spectrum of ^15^N-labeled Mth10b[M15QL19TV22Q] obtained using the procedure described in methods.

A preliminary study on the tertiary structure of Mth10b was carried out by using NMR. Due to the wild type Mth10b tending to aggregate at concentrations beyond 0.4 mM which results in seriously less signals than needed in three-dimensional spectra, such as ^15^N-^13^C CBCA(CO)NH, HNCACB, we tried to introduce some mutations in Mth10b to improve its quality for NMR study. Fortunately, a triple mutant Mth10b[M15QL19TV22Q], in which Met^15^, Leu^19^ and Val^22^ are replaced by Gln^15^, Thr^19^ and Gln^22^ respectively, was found no longer aggregate in higher concentrations and can give qualified three-dimensional spectra. Using the three-dimensional spectra of this triple mutant, we finished the backbone assignment. The 2D ^1^H-^15^N HSQC spectrum of the Mth10b variant Mth10b[M15QL19TV22Q], presented in Figure 5, shows a set of well-dispersed cross-peaks for almost all residues in the protein (each cross-peak represents a signal from a single N-H pair), indicating that the recombinant Mth10b has a well-folded tertiary structure. As shown in Figure 5, the chemical shifts of ^15^N resonances are distributed from approximately 107 ppm to 130 ppm, and the chemical shifts of ^1^H resonances are distributed from 6.5 ppm to 9.5 ppm. Due to the amino acid sequence of Mth10b sharing highly identity with its archaeal homologs, not surprisingly, those conserved residues in Mth10b and other typical Sac10b family members, such as Ssh10b [7], share highly similar distribution. Combining the facts that Mth10b shares high similarity with other typical Sac10b family members with respect to the primary structure and secondary structure, similar 2D ^1^H-^15^N HSQC spectrum suggest that the three-dimensional structure of Mth10b is similar to the three-dimensional structures of typical Sac10b family proteins.

### Mth10b exists as a dimer in solution

**Figure 6:**
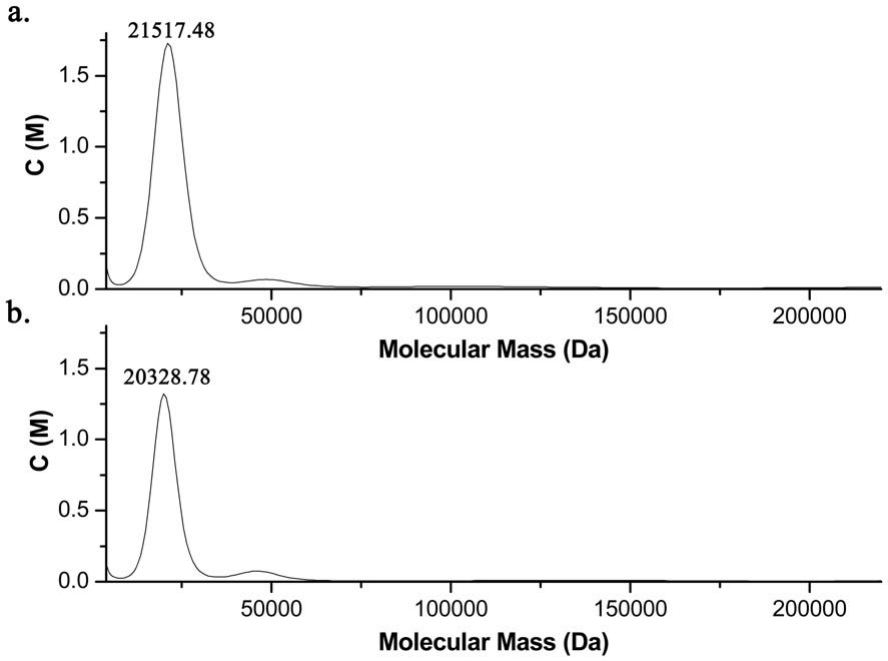
The apparent molecular mass of Mth10b in buffer F determined by analytical ultracentrifugation: a. at protein concentration of 1.0 mg/mL; b. at protein concentration of 0.6 mg/mL.

The association state of Mth10b in solution was analyzed by analytical ultracentrifugation. According to the experimental results, as shown in figure 6, the apparent molecular mass of Mth10b is 21.5 kDa and 20.3 kDa in buffer F at protein concentrations of 1.0 and 0.6 mg/mL, respectively. These values are close to the expected value of Mth10b dimer (19.7 kD), indicating that Mth10b exists primarily as a stable dimer in solution. Moreover, gel filtration analysis indicated that Mth10b is present predominantly as a dimer in solution (data not shown). These results indicate that Mth10b exists in solution in an oligomeric form that is similar to those typical members of the Sac10b protein family.

### Nucleic acid-binding affinity

**Figure 7:**
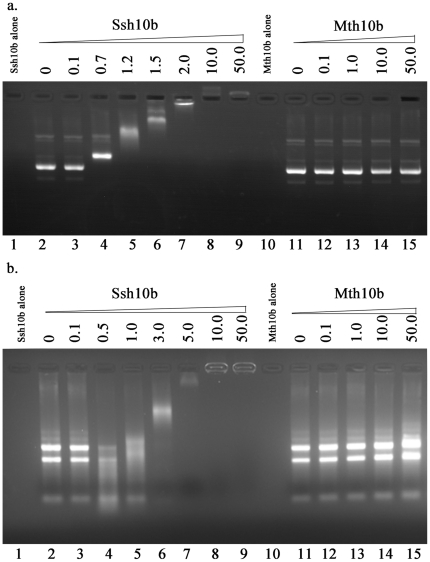
EMSA of nucleic acid binding by Mth10b and Ssh10b. a. DNA binding activity: 370 ng of plasmid DNA was incubated with increasing quantities of protein followed by electrophoresis on agarose gels. The protein quantities (µg) are marked on the lanes' top. Lane 1 and lane 10 represent 10 µg Ssh10b and Mth10b alone, respectively; b. RNA binding activity: 1.1 µg of total RNA of *Escherichia coli* was incubated with increasing quantities of protein followed by electrophoresis on agarose gels. The protein quantities (µg) are marked on the lanes' top. Lane 1 and lane 10 are 10 µg Ssh10b and Mth10b alone, respectively.

The nucleic acid-binding affinity of Mth10b was investigated by agarose gel electrophoresis using Ssh10b as a positive control. Increasing quantities of recombinant proteins were incubated with supercoiled plasmid pAS22 or total RNA from *Escherichia coli* DH5α in binding buffer, followed by separation of bound and free species using an agarose gel and visualization by ethidium bromide staining. Figure 7a shows the results of DNA-binding experiments: for Ssh10b, 370 ng of supercoiled plasmid DNA were visibly shifted at protein quantities greater than 0.7 µg. At higher quantities of Ssh10b, the plasmid DNA was progressively retarded until it was apparently saturated at recombinant protein levels greater than 2 µg and could not enter the gel. The plasmid DNA showed no significant band shift in samples incubated with Mth10b, even at protein quantities of 50 µg. Figure 7b shows the results of RNA-binding experiments: for Ssh10b, 1.1 µg of total RNA from *Escherichia coli* DH5α were visibly shifted at protein quantities greater than 1 µg. At higher quantities of Ssh10b, the RNA became progressively retarded until it apparently saturated the RNA at recombinant protein levels greater than 5 µg, preventing the RNA from entering the gel. The total RNA samples incubated with Mth10 showed no significant band shift, even at protein quantities of 50 µg. These results indicated that DNA and RNA can bind to Ssh10b with similar affinities *in vitro*, in agreement with previous reports [4]–[6], but that Mth10b can bind neither DNA nor RNA *in vitro*. These results distinguish Mth10b from other members of the Sac10b protein family, indicating that Mth10b may have a physiological function that is distinct from the functions of other members of the Sac10b protein family.

## Discussion

Although originally identified as DNA-binding proteins, there is evidence that Sac10b protein family members can also bind RNA. Using immunogold electron microscopy, Bohrmann *et al.* found that Sac10b is located exclusively in the cytoplasm rather than in the nucleus [3]. Although Ssh10b binds to DNA and RNA with similar affinities *in vitro* [5], [6], Guo *et al.* found that it binds exclusively to RNA *in vivo* using ultraviolet irradiation [6]. Using chromatin immunoprecipitation, Marsh *et al.* found that Sso10b is associated with all investigated chromosomal regions but was released from the insoluble chromatin-containing pellet by treatment with either DNase I or RNase A. This result indicated that Sso10b is associated with both DNA and RNA *in vivo* [17]. The overall structures of typical Sac10b protein family members are reminiscent of the C-terminal domain of bacterial translation initiation factor IF3 and the N-terminal domain of DNase I. Aravind *et al.* used a bioinformatics analysis to show that the Sac10b protein family is related to two eukaryotic protein families involved in RNA metabolism [29]. They suggested that highly conserved Sac10b homologs may have an additional or exclusive role in RNA metabolism, especially in organisms for which there is no evidence of a major chromosomal role [29]. In general, the exact physiological functions of Sac10b protein family members remain unclear, though considerable efforts have been invested in recent years. However, all previous studies presumed that the nucleic acid-binding ability of Sac10b family proteins was associated with their actual functions.

In this study, we identified Mth10b from *Methanobacterium thermoautotrophicum ΔH*, a unique member of the Sac10b protein family whose amino acid sequence shares high homology with other members of the Sac10b protein family. Interestingly, Mth10b has many more acidic amino acid residues than basic residues, and its isoelectric point is only 4.56 (Table 1), which is inconsistent with the characteristics of a nucleic acid-binding protein. We expressed Mth10b in *Escherichia coli* and purified the protein using a three-column chromatography purification procedure. Biochemical characterization indicated that Mth10b shares secondary, tertiary and even quaternary structure with typical members of the Sac10b protein family. However, EMSA showed that neither DNA nor RNA bound to Mth10b *in vitro*, a finding that distinguishes Mth10b from other members of the Sac10b protein family. Therefore, we hypothesize that either Mth10b likely has a physiological function that is distinct from the functions of most Sac10b family members or nucleic acid-binding ability may not be a fundamental factor to the actual function of the Sac10b family. The nature of this novel physiological function, however, is not known. Experiments to clarify the structure and actual function of Mth10b, combining crystallography, NMR and cell biology, are in progress.

## Materials and Methods

### Materials

The *Methanobacterium thermoautotrophicum ΔH* strain was obtained from the American Type Culture Collection (ATCC). The plasmid pET11a from Novagen was used to make the vector-DNA construct. *Escherichia coli* DH5α and BL21 (DE3) cells were used for plasmid cloning and protein expression, respectively. The expression plasmid pET11a-*ssh10b* containing the *ssh10b* gene was obtained from our laboratory stocks. Enzymes and reagents for DNA manipulations were purchased from TAKARA. Plasmid miniprep kits were obtained from OMEGA. Yeast extract and tryptone were purchased from OXOID. Isopropyl β-D-thiogalactoside (IPTG) was obtained from MERCK. ^15^N-labeled ammonium chloride and ^13^C-labeled glucose were purchased from Cambridge Isotope Laboratories, Inc. All chemicals were of analytical grade for biochemical use. All apparatus and chromatography materials were purchased from GE.

### Plasmid construction

The *mth10b* gene (ID code: MTH1483) was amplified from *Methanobacterium thermoautotrophicum ΔH* using polymerase chain reaction (PCR) with the forward primer 5′-TA**CATATG**TCAGAGGAGAATGTAG-3′ and reverse primer 5′-CC**GGATCC**TATTATTAATCCTTTCGGAGCTGA-3′. PCR introduced *NdeI* and *BamHI* restriction sites (underlined and in boldface) at the 5′ and 3′ ends, respectively, of the amplified fragment. The PCR product was digested with *NdeI* and *BamHI* and ligated into the expression plasmid pET11a, which was linearized with the same two enzymes. The constructed plasmid pET11a-*mth10b* was confirmed by DNA sequencing. Using the parental plasmid pET11a-*ssh10b*, the expression plaimid of the Mth10b variants were constructed. All mutations were introduced by site-directed mutagenesis through overlap extension PCR and verified by DNA sequencing.

### Protein expression and purification

Recombinant Ssh10b protein was expressed in the *Escherichia coli* BL21 (DE3) expression strain and purified as described previously [9].

For expression of Mth10b and its variants, the constructed plasmid pET11a-*mth10b* was also transformed into the *Escherichia coli* BL21 (DE3) expression strains. A single colony was picked and grown in 100 ml of LB media containing 100 µg/ml ampicillin with shaking (approximately 200 rpm) at 37°C overnight. The cultures were diluted 1∶50 in fresh antibiotic-containing LB media, and the cells were grown at 37°C until reaching an OD_600_ of 0.8. Protein expression was induced by the addition of IPTG at a final concentration of 0.3 mM and incubating at 25°C overnight. The cells were harvested by centrifugation at 4,000 rpm for 30 min.

For purification, the harvested cell pellets from 3-L cultures were resuspended in 75 ml of buffer A (20 mM Tris-HCl/pH 7.5) and disrupted by ultrasonication in ice bath. After centrifugation at 16,000 rpm for 30 min, the supernatant was loaded onto a 15-ml Q Sepharose Fast Flow column equilibrated with buffer A. The bound proteins were eluted with a 0–50% gradient (120 ml) of buffer B (1.5 M NaCl, 20 mM Tris-HCl/pH 7.5). Fractions containing Mth10b were identified by 15% SDS-PAGE and dialyzed against buffer A overnight. The sample was adjusted to a final concentration of 1 M (NH_4_)_2_SO_4_ by the addition of buffer C (3 M (NH_4_)_2_SO_4_, 20 mM Tris-HCl/pH 7.5). After centrifugation at 16,000 rpm for 30 min, the supernatant was loaded onto a 20-ml Phenyl Sepharose High Performance column that was equilibrated with buffer D (1 M (NH_4_)_2_SO_4_, 20 mM Tris-HCl/pH 7.5), and proteins were eluted with a 0–100% gradient (120 ml) of buffer A. Fractions containing the target protein, identified by SDS-PAGE, were dialyzed against buffer A overnight. After centrifugation at 16,000 rpm for 30 min, the dialyzed sample was loaded onto a 6-ml Resource-S column equilibrated with buffer A, and proteins were eluted with a 0–50% gradient (60 ml) of buffer B. Fractions containing the recombinant protein, identified by SDS-PAGE, were dialyzed against buffer E (50 mM NH_4_HCO_3_) and lyophilized. All chromatography experiments were performed on an AKTA Purifier-10 system.

### Far-UV CD measurements

The far-UV CD measurements were performed on a PiStar-180 spectrometer (Applied Photophysics Ltd, UK) at 25°C. The protein samples were prepared in buffer F (50 mM Na_2_HPO_4_-NaH_2_PO_4_/pH 7.5) at a concentration of 0.25 mg/ml. Measurements were carried out by using a rectangular quartz cuvette with a path-length of 1 mm over the 190- to 250-nm wavelength range with a 2-nm bandwidth. Each spectrum was the average of five scans and was corrected for spurious signals generated by the solvent.

### NMR spectroscopy

The uniformly ^15^N/^13^C-labeled proteins were expressed in *Escherichia coli* BL21 (DE3) in M9 minimal medium containing ^15^NH_4_Cl and ^13^C-glucose as the sole nitrogen and carbon sources, respectively. The labeled proteins were purified as described above. The purity of the protein was demonstrated by its visualization as a single band on SDS-PAGE and an ultraviolet absorbance ratio of A_280_/A_260_≧1.7. Samples for NMR measurements contained 1.0–2.0 mM labeled proteins, 90% H_2_O/10% D_2_O, 200 mM NaCl, 5 mM DTT, 0.02% NaN3, 1 mM EDTA and 0.01% sodium-2,2-dimethyl-2-silapentane-5-sulfomate (DSS) in 20 mM Tris-HCl buffer (pH 7.5).

All NMR experiments were carried out at 37°C on a Bruker Advance DMX 600 MHz spectrometer equipped with a triple resonance cryo-probe. Resonance assignments for backbone ^1^H^N^, ^15^N, ^13^C^α^ and ^13^C^β^ nuclei were indentified using 2D ^1^H-^15^N HSQC, 3D CBCA(CO)NH and HNCACB experiments. All NMR spectra were processed and analyzed using FELIX98 software (Accelrys Inc.). Proton chemical shifts and ^15^N and ^13^C chemical shifts were referenced to internal DSS and indirectly to DSS, respectively [30].

### Analytical ultracentrifugation

Sedimentation velocity experiments were performed on a Beckman-Coulter XL-A analytical ultracentrifuge using two-channel centerpieces with an An60Ti rotor at 60000 rpm and 20°C. Sedimentation was monitored using absorption at UV 238 nm with protein concentrations of 0.6 and 1.0 mg/mL in buffer F. UV absorption was scanned every 30 s for 6 h. Data were analyzed with software provided by Beckman Instruments (Palo Alto, CA).

### Nucleic acid binding

The nucleic acid-binding affinity of Mth10b was analyzed by EMSA using Ssh10b, a typical member of the Sac10b protein family, as a positive control. Approximately 370 ng of supercoiled plasmid pAS22 (3789 bp) or 1.1 µg total RNA from *Escherichia coli* DH5α cells were incubated with varying quantities of purified recombinant protein in buffer G (10 mM HEPES, 100 mM NaCl/pH 7.0) in a total volume of 15 µl at room temperature for 15 min. The samples were resolved by electrophoresis in 1.2% agarose gels in 1×TAE buffer at constant voltage. After electrophoresis, the gels were stained with ethidium bromide and visualized under UV light.

## References

[1] Grote M, Dijk J, Reinhardt R (1986). Ribosomal and DNA binding proteins of the thermoacidophilic archaebacterium *Sulfolobus acidocaldarius*.. Biochim Biophys Acta.

[2] Lurz R, Grote M, Dijk J, Reinhardt R, Dobrinski B (1986). Electron microscopic study of DNA complexes with proteins from archaebacterium *Sulfolobus acidocaldarius*.. EMBO J.

[3] Bohrmann B, Kellenberger E, Arnold-Schulz-Gahmen B, Sreenivas K, Suryanaranaya T (1994). Localization of histone-like proteins in thermophilic Archaea by immunogold electron microscopy.. J Struct Biol.

[4] Forterre P, Confalonieri F, Knapp S (1999). Identification of the gene encoding archeal-specific DNA-binding proteins of the Sac10b family.. Mol Microbiol.

[5] Xue H, Guo R, Wen Y, Liu D, Huang L (2000). An abundant DNA binding protein from the hyperthermophilic archaeon *Sulfolobus shibatae* affects DNA supercoiling in a temperature-dependent fashion.. J Bacteriol.

[6] Guo R, Xue H, Huang L (2003). Ssh10b, a conserved thermophilic archaeal protein, binds RNA *in vivo*.. Mol Microbiol.

[7] Cui Q, Tong Y, Xue H, Huang L, Feng Y (2003). Two conformations of Archaeal Ssh10b.. J Biol Chem.

[8] Xu S, Qin S, Pan XM (2004). Thermal and conformational stability of Ssh10b protein from archaeon *Sulfolobus shibattae*.. Biochem J.

[9] Ge M, Xia XY, Pan XM (2008). Salt bridges in the hyperthermophilic protein Ssh10b are resilient to temperature increases.. J Biol Chem.

[10] Mao YJ, Sheng XR, Pan XM (2007). The effects of NaCl concentration and pH on the stability of hyperthermophilic protein Ssh10b.. BMC Biochem.

[11] Fang X, Cui Q, Tong Y, Feng Y, Shan L (2008). A stabilizing alpha/beta-hydrophobic core greatly contributes to hyperthermostability of archaeal [P62A]Ssh10b.. Biochemistry.

[12] Fang X, Feng Y, Wang J (2009). Favorable contribution of the C-terminal residue K97 to the stability of a hyperthermophilic archaeal [P62A]Ssh10b.. Arch Biochem Biophys.

[13] Ge M, Mao YJ, Pan XM (2009). Refolding of the hyperthermophilic protein Ssh10b involves a kinetic dimeric intermediate.. Extremophiles.

[14] Ge M, Pan XM (2009). The contribution of proline residues to protein stability is associated with isomerization equilibrium in both native and unfolded states.. Extremophiles.

[15] Bell SD, Botting CH, Wardleworth BN, Jackson SP, White MF (2002). The interaction of Alba, a conserved archaeal chromatin protein, with Sir2 and its regulation by acetylation.. Science.

[16] Wardleworth BN, Russell RJ, Bell SD, Taylor GL, White MF (2002). Structure of Alba: an archaeal chromatin protein modulated by acetylation.. EMBO J.

[17] Marsh VL, Peak-Chew SY, Bell SD (2005). Sir2 and the acetyltransferase, Pat, regulate the archaeal chromatin protein Alba.. J Biol Chem.

[18] Chou CC, Lin TW, Chen CY, Wang AH (2003). Crystal structure of the hyperthermophilic archaeal DNA-binding protein Sso10b2 at a resolution of 1.85 Angstroms.. J Bacteriol.

[19] Biyani K, Kahsai MA, Clark AT, Armstrong TL, Edmondson SP (2005). Solution structure, stability, and nucleic acid binding of the hyperthermophile protein Sso10b2.. Biochemistry.

[20] Jelinska C, Conroy MJ, Craven CJ, Hounslow AM, Bullough PA (2005). Obligate heterodimerization of the archaeal Alba2 protein with Alba1 provides a mechanism for control of DNA packaging.. Structure.

[21] Jelinska C, Petrovic-Stojanovska B, Ingledew WJ, White MF (2010). Dimer-dimer stacking interactions are important for nucleic acid binding by the archaeal chromatin protein Alba.. Biochem J.

[22] Wang G, Guo R, Bartlam M, Yang H, Xue H (2003). Crystal structure of a DNA binding protein from the hyperthermophilic euryarchaeon *Methanococcus jannaschii*.. Protein Sci.

[23] Zhao K, Chai X, Marmorstein R (2003). Structure of a Sir2 substrate, Alba, reveals a mechanism for deacetylation-induced enhancement of DNA binding.. J Biol Chem.

[24] Kumarevel T, Sakamoto K, Gopinath SC, Shinkai A, Kumar P (2008). Crystal structure of an archaeal specific DNA-binding protein (Ape10b2) from *Aeropyrum pernix K1*.. Proteins.

[25] Hada K, Nakashima T, Osawa T, Shimada H, Kakuta Y (2008). Crystal structure and functional analysis of an archaeal chromatin protein Alba from the hyperthermophilic archaeon *Pyrococcus horikoshii OT3*.. Biosci Biotechnol Biochem.

[26] Smith DR, Doucette-Stamm LA, Deloughery C, Lee H, Dubois J (1997). Complete genome sequence of *Methanobacterium thermoautotrophicum Delta H*: functional analysis and comparative genomics.. J Bacteriol.

[27] Altschul SF, Gish W, Miller W, Myers EW, Lipman DJ (1990). Basic local alignment search tool.. J Mol Biol.

[28] Xuan J, Yao H, Feng Y, Wang J (2009). Cloning, expression and purification of DNA-binding protein Mvo10b from *Methanococcus voltae*.. Protein Expr Purif.

[29] Aravind L, Lakshminarayan MI, Anantharamana V (2003). The two faces of Alba: the evolutionary connection between proteins participating in chromatin structure and RNA metabolism.. Genome Biol.

[30] Markley JL, Bax A, Arata Y, Hilbers CW, Kaptein R (1998). Recommendations for the presentation of NMR structures of proteins and nucleic acids.. J Mol Biol.

